# Early-Term Neonates Demonstrate a Higher Likelihood of Requiring Phototherapy Compared to Those Born Full-Term

**DOI:** 10.3390/children10111819

**Published:** 2023-11-16

**Authors:** Teck-Jin Tan, Wan-Ju Chen, Wan-Chun Lin, Ming-Chun Yang, Ching-Chung Tsai, Yung-Ning Yang, San-Nan Yang, Hsien-Kuan Liu

**Affiliations:** 1 Department of Pediatrics, E-Da Hospital, I-Shou University, Kaohsiung 82445, Taiwan; ed112285@edah.org.tw (T.-J.T.); ed105451@edah.org.tw (M.-C.Y.); ed102514@edah.org.tw (C.-C.T.); ancaly@isu.edu.tw (Y.-N.Y.); ed107078@edah.org.tw (S.-N.Y.); 2 School of Medicine, College of Medicine, I-Shou University, Kaohsiung 82445, Taiwan; ed107686@edah.org.tw; 3 Department of Pediatrics, E-Da Dachang Hospital, I-Shou University, Kaohsiung 80794, Taiwan; 4 Department of Nurse Practitioners, Yuan’s General Hospital, Kaohsiung 80249, Taiwan; y6397@yuanhosp.com.tw

**Keywords:** early term, full term, hyperbilirubinemia, phototherapy, transcutaneous bilirubin

## Abstract

Early-term neonates (with a gestational age (GA) of 37 and 0/7 weeks to 38 and 6/7 weeks) face higher morbidities, including respiratory and neurodevelopmental issues, than full-term (39 and 0/7 weeks to 40 and 6/7 weeks) infants. This study explores whether hyperbilirubinemia necessitating phototherapy also differs between these groups. A retrospective study was conducted on neonates born from January 2021–June 2022, excluding those with specific conditions. Evaluated factors included GA, birth weight, bilirubin levels, glucose-6-phosphate dehydrogenase (G6PD) deficiency, and feeding type, with phototherapy given as per AAP guidelines. Of 1085 neonates, 356 met the criteria. When stratifying the neonates based on the need for phototherapy, a higher proportion of early-term neonates required phototherapy compared to full-term (*p* < 0.05). After factoring in various risks (GA; birth weight; gender; feeding type; G6PD deficiency; transcutaneous bilirubin levels at 24 h and 24–48 h postpartum; maternal diabetes; and the presence of caput succedaneum or cephalohematoma), early-term neonates were more likely to need phototherapy than full-term babies (OR: 2.15, 95% CI: 1.21 to 3.80). The optimal cut-off for transcutaneous bilirubin levels 24–48 h postpartum that were used to predict phototherapy need was 9.85 mg/dl. In conclusion, early-term neonates are at a greater risk for developing jaundice and requiring phototherapy than full-term neonates. Monitoring bilirubin 24–48 h postpartum enhances early prediction and intervention.

## 1. Introduction

Although early-term neonates (defined as those with a gestational age ranging from 37 and 0/7 weeks to 38 and 6/7 weeks) are categorized as term babies (with a gestational age of 37 and 0/7 weeks to 41 and 6/7 weeks), they manifest significantly higher probabilities of requiring admission to the neonatal intensive care unit, encountering respiratory morbidity, developing hypoglycemia, and needing intravenous fluids when compared to full-term neonates (ranging from 39 and 0/7 weeks to 40 and 6/7 weeks) [1,2,3]. Existing literature also indicates that longer gestation, even within term infants, is beneficial for both cognitive and motor development [4]. Given that early-term infants display inferior neurodevelopment, respiratory stability, and blood glucose stability, it raises the question of whether the levels of bilirubin, which requires hepatic metabolism, also differ when compared to full-term neonates.

Currently, the management and treatment of neonatal hyperbilirubinemia is determined according to guidelines set forth by the American Academy of Pediatrics (AAP), which account for gestational age (GA) and various risk factors [5,6]. If a neonate’s bilirubin level is in the high-risk zone according to the hour-specific bilirubin nomogram, appropriate treatment (phototherapy or exchange transfusion) is administered, following AAP guidelines, to neonates with a GA of ≥35 weeks [5].

Considering the differences in postnatal morbidity and neurodevelopmental outcomes between early-term and full-term neonates, exploring the disparities in significant hyperbilirubinemia is noteworthy. Previous studies have indicated that early-term infants have a higher likelihood of requiring phototherapy compared to full-term neonates [7,8]. However, most of these studies were epidemiological in nature and did not analyze common risk factors for hyperbilirubinemia. Therefore, this study aims to determine, after accounting for certain hyperbilirubinemia risk factors, whether there is still a significant difference in the rates of phototherapy between early-term and full-term infants.

## 2. Materials and Methods

### 2.1. Study Design and Subjects

This research comprised a retrospective examination conducted at the E-Da Hospital, Kaohsiung, Taiwan. The scope of patient-related data spanned a temporal window from January 2021 through to June 2022. This study has been approved by the Institutional Review Board of E-Da Hospital, under the reference number EMRP30111N (date of approval: 4 August 2022). All patient data were de-identified prior to statistical analysis, ensuring the preservation of patient confidentiality. Neonates born at E-Da Hospital from January 2021 to June 2022 were initially included in the study, followed by subsequent exclusions. We reviewed the medical records of these patients and organized the data regarding basal characteristics (GA, gender, Cesarean section or vaginal delivery, birth weight); levels of transcutaneous bilirubin (TcB) within 24 h postpartum and between 24 and 48 h postpartum; the presence of G6PD deficiency; the type of feeding (exclusive breastfeeding or not); maternal diabetes; and the presence of caput succedaneum or cephalohematoma. Additionally, we recorded whether the neonates subsequently underwent phototherapy. Furthermore, delayed cord clamping is not practiced for term babies in our hospital. Therefore, the bilirubin level in this cohort is not influenced by this procedure. After data curation, the participants were categorized based on 1. whether the GA was ≤38 and 6/7 weeks or ≥39 and 0/7 weeks, and 2. whether they had received phototherapy thereafter.

### 2.2. The Timing for Determining Whether to Administer Phototherapy

At our institution, we use the JM-103 Transcutaneous Bilirubinometer (Konica Minolta, Osaka, Japan) for TcB assessments of all neonates. This device undergoes regular calibration and maintenance. Bilirubin monitoring begins within the first 24 h after birth, with a subsequent measurement taken between 24 and 48 h postpartum. Daily assessments continue until the neonate is discharged. All results are meticulously recorded in the medical records. The medical personnel conducting these measurements have received hands-on training, and measurements are consistently taken from the neonate’s sternum area. Clinically, if TcB readings reach 70% of the phototherapy intervention threshold, a confirmatory serum total bilirubin blood test is performed [9]. We then reference these serum levels, and, in alignment with the AAP guidelines for hyperbilirubinemia in neonates with a GA of over ≥35 weeks, decisions are made about phototherapy treatment, factoring in GA and any existing risk factors [5].

### 2.3. Types of Neonatal Feeding

E-Da Hospital proudly holds national accreditation as a baby-friendly institution. In alignment with this distinction, we ardently advocate for mothers to initiate exclusive breastfeeding for their neonates right from the moment of birth. The introduction of formula supplementation is only contemplated under specific circumstances. These situations include the mother’s request for the use of formula milk; the occurrence of crystalluria in the neonate; a weight decrement that surpasses 10% of the initial birth weight; or instances where the mother is on a medication regimen that is deemed incompatible with breastfeeding. Following our protocols, neonates who are administered formula in tandem with breast milk are categorically characterized as not having been breastfed exclusively.

### 2.4. Statistical Analysis

We conducted statistical analysis on the organized data using SPSS version 20 (IBM Corp., Armonk, NY, USA). Proportions were utilized to present categorical variables, and means ± standard deviations were employed for continuous variables. The Mann–Whitney U test was used for the comparison of nonparametric continuous variables between groups, and Student’s *t*-test was employed for the comparison of parametric continuous variables between groups. The chi-square test was employed for comparing categorical variables. Additionally, we implemented multivariate logistic regression to adjust potential risk factors (gestational age, birth weight, gender, feeding type, G6PD deficiency, TcB at <24 h after birth and between 24–48 h after birth, maternal diabetes, and presence of caput succedaneum or cephalohematoma) associated with phototherapy. Moreover, a receiver operating characteristic (ROC) curve was plotted to determine the optimal threshold for predicting the probability of phototherapy requirement, depending on TcB levels between 24 and 48 h postpartum.

## 3. Results

**Figure 1:**
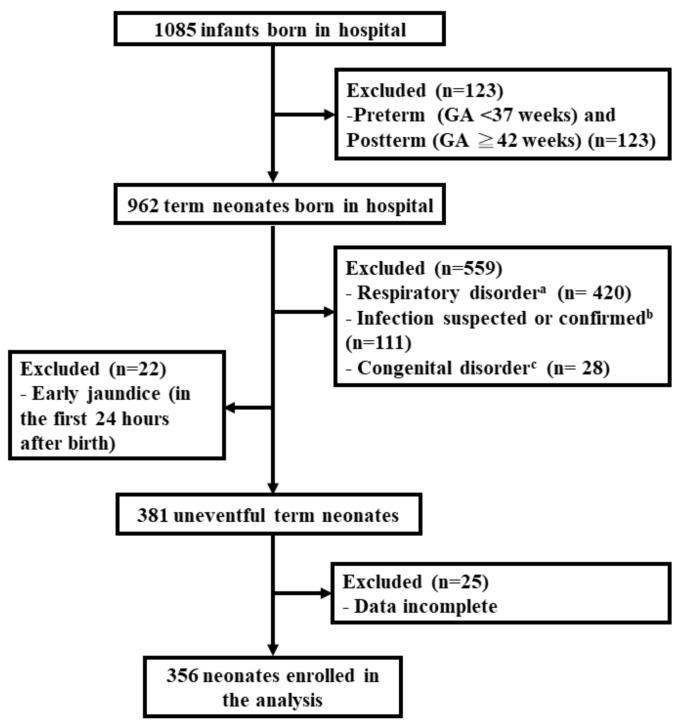
Neonate selection process from an initial cohort of 1085, following exclusion criteria application. ^a^ Encompasses transient tachypnea of the newborn, neonatal pneumonia/pneumonitis, and meconium aspiration syndrome; ^b^ encompasses fever episodes, bacteremia, and meningitis; ^c^ encompasses congenital anomalies of the heart, gastrointestinal tract, biliary tract, and additional genetic diseases.

Between 2021 and 2022, our institution recorded the births of 1085 neonates. From this population, neonates born preterm (GA < 37 and 0/7 weeks) and postterm (GA ≥ 42 and 0/7 weeks) were excluded (*n* = 123), as were neonates requiring postnatal hospitalization for respiratory conditions, including transient tachypnea of the newborn, neonatal pneumonia or pneumonitis, and meconium aspiration syndrome (*n* = 420). Cases with either suspected or confirmed infections were omitted (*n* = 111). Additionally, neonates presenting with congenital anomalies (e.g., congenital cyanotic heart disease or genetic anomalies, *n* = 28) and those who developed early onset jaundice (within the first 24 h postpartum, *n* = 22) were also excluded. After the exclusion of cases with incomplete data, 356 neonates were ultimately incorporated into this study for subsequent analysis (Figure 1). From this cohort, the average GA was computed to be 38.82 ± 0.87 weeks, and the mean birth weight was ascertained at 3091.32 ± 320.99 g. Furthermore, an observation from our dataset revealed that 135 out of the 356 neonates, equivalent to 37.9%, were exclusively breastfed. Concurrently, we identified that 16 neonates, constituting 4.49% of our study cohort, were diagnosed with glucose-6-phosphate dehydrogenase (G6PD) deficiency.

### 3.1. Differences between Neonates with a Gestational Age of ≤38 and 6/7 Weeks and Those ≥39 and 0/7 Weeks

We stratified the neonates enrolled in this study into two groups based on their GA: those ≤38 and 6/7 weeks, and those ≥39 and 0/7 weeks, in line with the definitions of early-term and full-term neonates. We observed a significant difference in birth weight between the two groups (3043.72 ± 327.78 g for ≤38 and 6/7 weeks vs. 3143.99 ± 305.71 g for ≥39 and 0/7 weeks, *p* < 0.05). Furthermore, the proportion of Cesarean sections was significantly higher in the ≤38 and 6/7 weeks group (79/187, 40.6%) compared to the ≥39 and 0/7 weeks group (15/169, 8.9%, *p* < 0.05). The incidence of neonates diagnosed with glucose-6-phosphate dehydrogenase deficiency was higher in the full-term group than in the early-term group. Additionally, the likelihood of receiving phototherapy was noticeably higher in the early-term group (59/187, 31.6%) than in the full-term group (36/169, 21.3%, *p* < 0.05). Other factors, such as gender; type of feeding (whether exclusive breastfeeding or not); levels of TcB within the initial 24 h and between 24 and 48 h postpartum; maternal diabetes; presence of caput succedaneum or cephalohematoma; and the trait of being small for gestational age did not demonstrate any significant difference between the two groups (Table 1).

### 3.2. Comparison between Groups Who Have Previously Received Phototherapy and Those Who Have Not

Upon categorizing the neonates according to whether they received phototherapy or not, significant differences emerged. When comparing GA, there was no significant difference between the non-phototherapy and phototherapy groups (38.88 ± 0.87 vs. 38.68 ± 0.87, *p* = 0.06). However, when stratified by early-term and full-term neonates, early-term infants had a significantly higher rate of phototherapy compared to full-term infants (59/95 (62.1%) vs. 36/95 (37.9%), *p* < 0.05). Additionally, a significantly higher prevalence of G6PD deficiency was observed in the phototherapy group. Further analysis revealed marked differences in TcB levels within 24 h postpartum (4.67 ± 1.79 vs. 5.79 ± 1.98 mg/dL, *p* < 0.05) and 24 to 48 h postpartum (8.30 ± 1.83 vs. 10.33 ± 1.87 mg/dL, *p* < 0.05). In contrast, other variables such as gender, birth weight, mode of delivery, maternal diabetes, feeding types, presence of caput succedaneum or cephalohematoma, and the trait of being small for gestational age showed no significant differences between the groups (Table 2).

### 3.3. Multivariable Logistic Regression Analysis for Determining Factors Associated with the Administration of Phototherapy

We employed logistic regression to analyze variables potentially impacting the likelihood of receiving phototherapy. In univariate analyses, factors such as GA (≤38 and 6/7 weeks vs. 39 and 0/7 weeks), the presence of glucose-6-phosphate dehydrogenase deficiency, and levels of TcB—within 24 h and between 24 and 48 h postpartum—were associated with an increased probability of undergoing phototherapy (*p* < 0.05). However, when subjected to multivariable logistic regression, only GA (adjusted odds ratio 2.15, 95% CI: 1.21 to 3.80) and TcB levels between 24 and 48 h postpartum (adjusted odds ratio 2.04, 95% CI: 1.65 to 2.52) exhibited significant variations (Table 3).

### 3.4. Evaluating the Diagnostic Value of TcB Levels between 24 and 48 h Postpartum in Predicting the Necessity of Phototherapy

**Figure 2:**
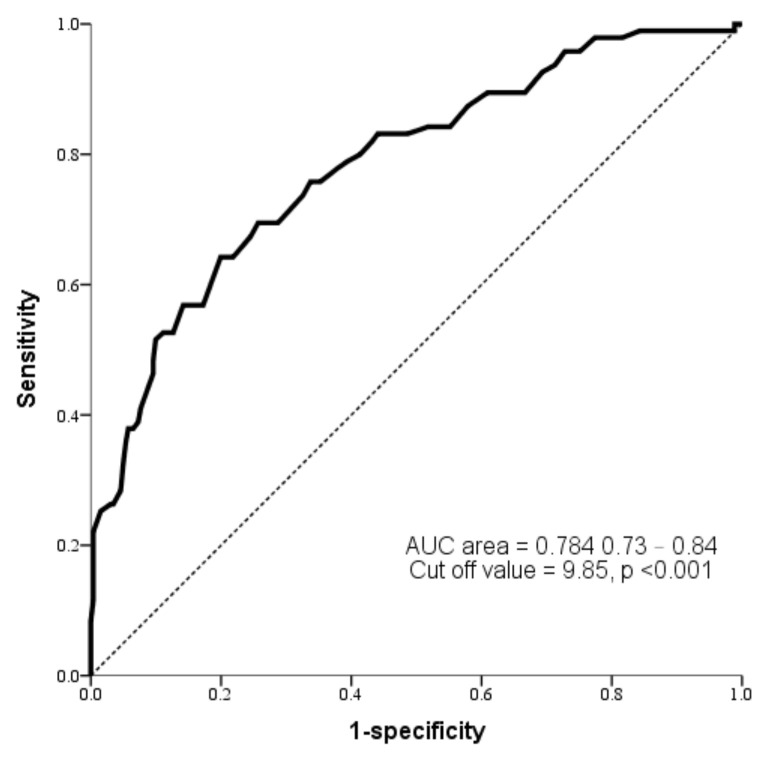
ROC curves of TcB levels measured between 24 and 48 h postpartum to predict the requirement for phototherapy.

Figure 2 illustrates the ROC curves of TcB levels measured between 24 and 48 h postpartum in order to predict the requirement of phototherapy. The area under the ROC curve reached 0.784 (95% CI [0.73–0.84], *p* < 0.001), signifying the necessity for phototherapy. The optimal cut-off value—established by the Youden index—for TcB levels 24 to 48 h postpartum to be used to identify phototherapy necessity was pinpointed at 9.85 mg/dL. Subsequently, we categorized the TcB levels between 24 and 48 h postpartum with 9.85 mg/dL serving as the cut-off point. The odds ratios associated with TcB levels >9.85 mg/dL between 24 and 48 h postpartum were 7.21 (95% CI [4.30–12.10], *p* < 0.001).

## 4. Discussion

Historically, a great deal of research has been devoted to distinguishing between term and preterm infants. Previous studies highlight that neonates born prematurely (defined as those with a GA of less than 37 weeks) exhibit significantly higher risks of mortality and numerous negative neonatal outcomes compared to their term counterparts (those with a GA of 37 weeks or more). Such adverse outcomes include extended stays in the neonatal intensive care unit; hyperbilirubinemia; episodes of apnea or bradycardia; sepsis onset; respiratory complications; and hypoglycemia, to name a few [10,11]. However, the term “term neonates” is a broad categorization and can be further split into early-term and full-term infants. The academic literature seems to have marginally overlooked the detailed distinctions between these two subgroups of term neonates, indicating a potential gap in our comprehensive knowledge.

Compared to full-term neonates, early-term infants—due to their immature physiological development—are often discussed alongside late preterm infants in clinical discussions. Previous research has highlighted that both of these groups (late preterm and early-term infants) exhibit an increased risk of respiratory issues; higher rates of admission to neonatal intensive care units; reduced cognitive abilities; and poorer academic outcomes when compared to full-term neonates. Moreover, both groups have been shown to have a considerably higher risk of mortality, as supported by numerous studies [12,13,14,15,16].

Significant hyperbilirubinemia in neonates is a pressing concern. The primary apprehension is the potential for untreated significant hyperbilirubinemia in a neonate to progress to kernicterus, leading to permanent neurological complications [17]. Currently, with careful bilirubin monitoring and the widespread use of phototherapy, the incidence of kernicterus in Taiwan has been progressively declining [17].

Within the literature on significant hyperbilirubinemia, comparisons between early-term and full-term neonates are relatively sparse. Existing research consistently indicates that late preterm neonates present with a heightened incidence of significant hyperbilirubinemia relative to their term counterparts [18,19]. However, the subtle distinctions between early-term and full-term neonates have not been extensively studied in this context. Our investigation illuminates that neonates with GAs of 37 and 0/7 weeks to 38 and 6/7 weeks experienced a notably increased likelihood of postnatal phototherapy than those with GAs of 39 and 0/7 weeks to 40 and 6/7 weeks, with the risk being amplified 2.12-fold. Furthermore, our data suggest that a TcB threshold of 9.85 mg/dl, measured within the 24–48 h postnatal window, effectively predicts the impending need for phototherapy in neonates.

Several prior studies have ventured into the domain of hyperbilirubinemia, typically drawing distinctions between early-term and full-term neonates. One notable study aligns with our observations, highlighting that early-term neonates showed a heightened likelihood of needing phototherapy compared to those with a GA of more than 39 weeks [20]. However, this study was primarily an epidemiological comparison and did not account for several established factors influencing neonatal hyperbilirubinemia, such as birth weight, the choice between exclusive breastfeeding and formula feeding, and the presence of G6PD deficiency [21,22,23,24,25,26]. In contrast, our study comprehensively integrated these well-documented risk factors into our analysis, potentially providing a more rounded and detailed insight into the subject.

There is another study that thoroughly examined TcB levels during the initial postnatal month for both early-term and full-term neonates [7]. This research precisely developed an hour-specific TcB nomogram covering the full initial postnatal month, tailored for both early-term and full-term neonates. Analyzing data from this nomogram revealed that neonates born during the early term consistently exhibited higher average TcB levels throughout the first month compared to their full-term counterparts [7]. This study’s results align closely with our findings, further highlighting the increased likelihood of early-term infants requiring phototherapy. However, a limitation to note is that this study, like the prior one, did not comprehensively consider several established risk factors. This omission might limit the scope and depth of their conclusions.

Moreover, another study mentioned that early-term neonates have a higher rate of respiratory morbidity and require phototherapy within 72 h postpartum compared to full-term neonates [8]. In this investigation, the authors segregated the enrolled neonates into early-term and full-term categories for comparative analysis. However, this research utilized maternal age and mode of delivery as factors for adjusted logistic regression. Like the previous two articles, the authors did not account for other common factors that might lead to neonatal hyperbilirubinemia, potentially rendering their results less convincing.

In our pursuit to create an early predictive model for significant hyperbilirubinemia necessitating phototherapy in term neonates, inclusive of both early-term and full-term infants, we extensively examined various indicators. Several works in the past have also tried to identify early predictors that could efficiently earmark neonates with an elevated risk of needing phototherapy. Potential markers explored in these studies include initial cord bilirubin concentrations; the relative ratio of TcB to total serum bilirubin in conjunction with vital clinical parameters, like GA; and sophisticated machine learning models that synthesize data points—like bilirubin concentrations, neonatal weight, GA, and hours elapsed since birth—for predictive purposes [27,28,29].

Our data also indicate that TcB levels surpassing 9.85 mg/dL between 24–48 h postpartum markedly elevate the probability of the neonate subsequently undergoing phototherapy. One study corroborates our conclusion, indicating that bilirubin measurements taken at the 24th and 48th hours postpartum serve as robust predictors for significant hyperbilirubinemia [30]. Nevertheless, in clinical practice, obtaining bilirubin measurements at precise times can be challenging for healthcare professionals. Hence, we propose using bilirubin levels measured within the 24–48 h window postpartum, providing healthcare providers with a more convenient and flexible timeframe.

Additionally, it is worth discussing that our study adhered to the guideline published by AAP in 2009. However, this guideline was updated in September 2022 [31]. In the new Clinical Practice Guideline, the distribution of the hour-specific nomogram is much more detailed in terms of gestational age and the presence or absence of risk factors. In this new guideline, the bilirubin levels requiring phototherapy for GAs from 35 to 40 weeks are individually plotted on an hour-specific nomogram. Within this updated hour-specific nomogram, we can observe that, for the same post-natal hours, neonates with a GA of 39 and 0/7 weeks to 40 and 6/7 weeks have a higher threshold for phototherapy compared to those with a GA of 37 and 0/7 weeks to 38 and 6/7 weeks. Consequently, our research seems to resonate with this new guideline for hyperbilirubinemia management. Furthermore, based on this new therapeutic guideline, the incidence and timing of phototherapy required for neonatal hyperbilirubinemia in future cases might differ from current study findings. This discrepancy presents a significant area for exploration. Therefore, this can be a direction where our team focuses our future research efforts.

We acknowledge some limitations in our research. First, our study was based on a retrospective analysis from a single center with a relatively limited sample size. Also, most neonates in our hospital are of Asian ethnicity, which is recognized to have a higher incidence of hyperbilirubinemia. Consequently, differences among various ethnicities might not be evident. Secondly, the measurement of TcB was not consistently performed by the same individual. Furthermore, various studies have examined the accuracy of TcB measurements on the forehead versus the sternum, yielding differing results [32,33]. In our institution, all medical personnel conducting the measurements have undergone rigorous training, and the measurement location is consistently at the neonate’s sternum area. As such, we believe the TcB values obtained in our study are reasonably reliable. Moreover, we recognize that Bilitool is an exceptionally user-friendly and reliable method to determine whether neonates require phototherapy. There are also numerous studies that have innovated new mobile application tools to monitor neonatal hyperbilirubinemia and determine the timing for receiving phototherapy, with highly accurate results [34]. However, most of these tools necessitate an internet connection to yield results. Our research primarily focuses on the utilization of the straightforward and long-standing clinical practice of using the transcutaneous bilirubinometer to monitor neonatal jaundice. This approach is quick, simple, and only requires a single device. With appropriate training, nearly any member of medical staff can execute it without the need for internet access or mobile electronic devices. Therefore, it presents a more convenient and feasible option for most hospitals. Lastly, there are factors, such as albumin, that might influence any decisions regarding phototherapy; these were not accounted for as risk factors in our study. Nevertheless, our regression model incorporated common variables, such as GA, weight, G6PD deficiency, and type of feeding. Thus, our study offers a more comprehensive analysis than prior investigations.

## 5. Conclusions

Compared to full-term neonates, early-term neonates have a higher likelihood of requiring phototherapy due to significant hyperbilirubinemia. Additionally, although transcutaneous bilirubin concentration is not as accurate as serum bilirubin, if it surpasses 9.85 mg/dl within the 24 to 48 h postnatal window, it serves as a strong predictive marker for the potential requirement of phototherapy among these neonates. In situations where serum bilirubin measurements might be unavailable or impractical, TcB readings taken 24–48 h after birth could provide valuable guidance for treatment decisions.

## Figures and Tables

**Table 1:** Comparison between groups with gestational age ≤38 and 6/7 weeks (early term) and ≥39 and 0/7 weeks (full term).

	Early Term (*n* = 187)	Full-Term (*n* = 169)	*p* *
GA ^a^ (weeks)	38.13 ± 0.53	39.59 ± 0.42	<0.05
Sex			0.83
Boy	93 (49.7%)	82 (48.5%)	
Girl	94 (50.3%)	87 (51.5%)	
Birth weight	3043.72 ± 327.78	3143.99 ± 305.71	<0.05
Mode of delivery			<0.05
Vaginal delivery	111 (59.4%)	154 (91.1%)	
Cesarean section	76 (40.6%)	15 (8.9%)	
Feeding			0.52
Exclusive breastfeeding	68 (36.4%)	67 (39.6%)	
Non-exclusivebreastfeeding	119 (63.6%)	102 (60.4%)	
Mothers requested	90 (48.1%)	85 (50.3%)	
Crystalluria	11 (5.9%)	9 (5.3%)	
Weight loss exceeding 10% of the initial birth weight	13 (6.9%)	6 (3.6%)	
Currently taking medications not recommended for breastfeeding	5 (2.7%)	2 (1.2%)	
G6PD ^b^ deficiency			<0.05
Yes	4 (2.1%)	12 (7.1%)	
No	183 (97.9%)	157 (92.9%)	
TcB ^c^ level (<24 h)	5.04 ± 1.89	4.90 ± 1.92	0.49
TcB ^c^ level (24–48 h)	8.80 ± 2.04	8.89 ± 2.06	0.70
Small for gestational age			0.13
Yes	29 (15.5%)	17 (10.1%)	
No	158 (84.5%)	152 (89.9%)	
Received phototherapy			<0.05
Yes	59 (31.6%)	36 (21.3%)	
No	128 (68.4%)	133 (78.7%)	
Maternal diabetes			0.09
Yes	19 (10.2%)	9 (5.3%)	
No	168 (89.8)	160 (94.7)	
Caput succedaneum or cephalohematoma			0.34
Yes	7 (3.7%)	10 (5.9%)	
No	180 (96.3%)	159 (94.1%)	

* *p* value was analyzed using Student’s *t*-test; ^a^ GA: gestational age; ^b^ G6PD: glucose-6-phosphate dehydrogenase; ^c^ TcB: transcutaneous bilirubin.

**Table 2:** Comparative analysis of neonates: non-phototherapy versus phototherapy groups.

	Non-Phototherapy (*n* = 261)	Phototherapy (*n* = 95)	*p* *
GA ^a^ (weeks)	38.88 ± 0.87	38.68 ± 0.87	0.06
GA ^a^			<0.05
≤38 and 6/7 weeks (early term)	128 (49.0%)	59 (62.1%)	
≥39 and 0/7 weeks (full term)	133 (51.0%)	36 (37.9%)	
Sex			0.40
Boy	132 (50.6%)	43 (45.3%)	
Girl	129 (49.4%)	52 (54.7%)	
Birth weight	3096.53 ± 325.54	3077.00 ± 309.35	0.60
Mode of delivery			0.84
Vaginal delivery	195 (74.7%)	70 (73.7%)	
Cesarean section	66 (25.3%)	25 (26.3%)	
Feeding type			0.14
Exclusive breastfeeding	105 (40.2%)	30 (31.6%)	
Non-exclusive breastfeeding	156 (59.8%)	65 (68.4%)	
Mothers requested	123 (47.1%)	52 (54.7%)	
Crystalluria	16 (6.1%)	4 (4.2%)	
Weight loss exceeding 10% of the initial birth weight	13 (5.1%)	6 (6.3%)	
Currently taking medications not recommended for breastfeeding	4 (1.5%)	3 (3.2%)	
G6PD ^b^ deficiency			<0.05
Yes	8 (3.1%)	8 (8.4%)	
No	253 (96.9%)	87 (91.6%)	
Small for gestational age			0.42
Yes	36 (13.8%)	10 (10.5%)	
No	225 (86.2%)	85 (89.5%)	
TcB ^c^ level (<24 h)	4.67 ± 1.79	5.79 ± 1.98	<0.05
TcB ^c^ level (24–48 h)	8.30 ± 1.83	10.33 ± 1.87	<0.05
Maternal diabetes			0.83
Yes	21 (8.0%)	7 (7.4%)	
No	240 (92%)	88 (92.6%)	
Caput succedaneum or cephalohematoma			0.80
Yes	12 (4.6%)	5 (5.3%)	
No	249 (95.4%)	90 (94.7%)	

* *p* value was analyzed using Student’s *t*-test; ^a^ GA: gestational age; ^b^ G6PD: glucose-6-phosphate dehydrogenase; ^c^ TcB: transcutaneous bilirubin.

**Table 3:** Multivariable logistic regression analysis to identify factors associated with phototherapy administration.

	Crude		Adjusted ^e^	
Variables	B (95% CI)	p *	B (95% CI)	p *
GA ^a^ (≤38 and 6/7 weeks vs. ≥39 and 0/7 weeks)	1.70 (1.05–2.75)	<0.05	2.15 (1.21–3.80)	<0.05
Birth weight	1.00 (0.99–1.00)	0.61	1.00 (0.99–1.00)	0.27
Gender	0.81 (0.50–1.30)	0.38	0.81 (0.46–1.43)	0.46
Feeding type ^c^	0.69 (0.42–1.13)	0.14	0.66 (0.37–1.18)	0.15
G6PD ^b^ deficiency	0.34 (0.13–0.94)	<0.05	0.56 (0.17–1.80)	0.33
TcB ^d^ level (<24 h)	1.37 (1.20–1.57)	<0.05	0.92 (0.77–1.10)	0.37
TcB ^d^ level (24–48 h)	1.89 (1.60–2.23)	<0.05	2.04 (1.65–2.52)	<0.05
Maternal diabetes	0.91 (0.37–2.21)	0.83	1.06 (0.29–3.93)	0.93
Caput succedaneum or cephalohematoma	1.15 (0.40–3.36)	0.80	0.86 (0.32–2.33)	0.77

* *p* value was analyzed using Student’s *t*-test; ^a^ GA: gestational age; ^b^ G6PD: glucose-6-phosphate dehydrogenase; ^c^ feeding type: exclusive breastfeeding versus non-exclusive breastfeeding; ^d^ TcB: transcutaneous bilirubin; ^e^ adjusted covariates: GA, birth weight, gender, feeding type, G6PD deficiency, TcB level at 24 h and 24–48 h after birth, maternal diabetes, and caput succedaneum or cephalohematoma.

## Data Availability

The data from this study can be obtained by reaching out to the corresponding author. They are not accessible to the public due to the private details of the participants they contain.
